# Detection of an IMI-2 carbapenemase-producing *Enterobacter asburiae* at a Swedish feed mill

**DOI:** 10.3389/fmicb.2022.993454

**Published:** 2022-10-21

**Authors:** Stefan Börjesson, Michael S. M. Brouwer, Emma Östlund, Jenny Eriksson, Josefine Elving, Oskar Karlsson Lindsjö, Linda I. Engblom

**Affiliations:** ^1^Department of Animal Health and Antimicrobial Strategies, National Veterinary Institute (SVA), Uppsala, Sweden; ^2^Department of Microbiology, Public Health Agency of Sweden, Solna, Sweden; ^3^Department of Laboratory Medicine, Karolinska Institute, Stockholm, Sweden; ^4^Department of Bacteriology, Host-Pathogen Interactions and Diagnostics Development, Wageningen Bioveterinary Research, Lelystad, Netherlands; ^5^Department of Microbiology, National Veterinary Institute (SVA), Uppsala, Sweden; ^6^Department of Chemistry, Environment and Feed Hygiene, National Veterinary Institute (SVA), Uppsala, Sweden

**Keywords:** antimicrobial resistance, plasmid, carbapenem resistance, *bla*_IMI-2_, environment, *Enterobacter cloacae* complex, clinical isolates

## Abstract

Occurrence of multidrug resistant Enterobacteriaceae in livestock is of concern as they can spread to humans. A potential introduction route for these bacteria to livestock could be animal feed. We therefore wanted to identify if *Escherichia* spp., *Enterobacter* spp., *Klebsiella* spp., or *Raoutella* spp. with transferable resistance to extended spectrum cephalosporins, carbapenems or colistin could be detected in the environment at feed mills in Sweden. A second aim was to compare detected isolates to previous described isolates from humans and animals in Sweden to establish relatedness which could indicate a potential transmission between sectors and feed mills as a source for antibiotic resistant bacteria. However, no isolates with transferable resistance to extended-cephalosporins or colistin could be identified, but one isolate belonging to the *Enterobacter cloacae* complex was shown to be carbapenem-resistant and showing carbapenemase-activity. Based on sequencing by both short-read Illumina and long-read Oxford Nanopore MinIon technologies it was shown that this isolate was an *E. asburiae* carrying a *bla*_IMI-2_ gene on a 216 Kbp plasmid, designated pSB89A/IMI-2, and contained the plasmid replicons IncFII, IncFIB, and a third replicon showing highest similarity to the IncFII(Yp). In addition, the plasmid contained genes for various functions such as plasmid segregation and stability, plasmid transfer and arsenical transport, but no additional antibiotic resistance genes. This isolate and the pSB89A/IMI-2 was compared to three human clinical isolates positive for *bla*_IMI-2_ available from the Swedish antibiotic monitoring program Swedres. It was shown that one of the human isolates carried a plasmid similar with regards to gene content to the pSB89A/IMI-2 except for the plasmid transfer system, but that the order of genes was different. The pSB89A/IMI-2 did however share the same transfer system as the *bla*_IMI-2_ carrying plasmids from the other two human isolates. The pSB89A/IMI-2 was also compared to previously published plasmids carrying *bla*_IMI-2_, but no identical plasmids could be identified. However, most shared part of the plasmid transfer system and DNA replication genes, and th*e bla*_IMI-2_ gene was located next the transcription regulator *imi*R. The IS3-family insertion element downstream of *imi*R in the pSB89A was also related to the IS elements in other *bla*_IMI_-carrying plasmids.

## Introduction

Antibiotic resistant bacteria are one of the major threats to modern human and animal health care worldwide. Of major concern is the increasing trends of extended spectrum beta-lactamases (ESBL) and, plasmid-mediated AmpC β-lactamase (pAmpC)-producing Enterobacteriaceae and carbapenem-resistant Enterobacteriaceae (CRE) in both humans and animals (Bezabih et al., 2020; Hansen, 2021). In addition, these bacteria also occur in the environment, including wildlife, but the occurrence appears mainly to mirror the one described in human populations (Guenther et al., 2011; Atterby et al., 2017). Due to increasing trends of human infections caused by multidrug-resistant CRE, the antibiotic colistin, previously used mainly in food-producing animals, has received renewed attention as a treatment of human infections (Falagas et al., 2011). Resistance to colistin was previously thought only to be connected to chromosomal mutations, and despite the relatively extensive use in livestock settings, the proportion of colistin-resistance among Enterobacteriaceae from healthy animals appeared to remain low (Kempf et al., 2013). However, this changed in 2015 when increasing trends of colistin-resistant Enterobacteriaceae due to a transferable colistin resistance gene *mcr*-1 was described in China (Liu et al., 2016). Since the description of *mcr*-1 an additional nine *mcr*-genes have been described and occurrence has been shown in humans, animals, and the environment worldwide (Elbediwi et al., 2019).

The occurrence of ESBL- and pAmpC-producing Enterobacteriaceae, CRE and Enterobacteriaceae with *mcr*-genes in animals, primarily livestock, is of concern as animal populations can function as reservoirs for occurrence in humans, and transmissions from animals to humans have been indicated (Börjesson et al., 2016; Madec et al., 2017). The environment may also be a reservoir and dissemination routes for these resistant bacteria (EFSA, 2021). There also exists a risk that new strains of ESBL-, pAmpC-producing Enterobacteriaceae and CRE can emerge in the environment and bacteria may acquire previously unknown genes encoding antibiotic resistance in the environment. For example, the *bla*_CTX-M_ encoding ESBL and *bla*_OXA-48_ encoding carbapenemase appear to be a progeny from genes in the environmental bacteria *Kluyvera* spp. and *Shewanella* spp., respectively (Humeniuk et al., 2002; Tacão et al., 2018).

Despite Sweden having a relativity low incidence of ESBL-, pAmpC-producing Enterobacteriaceae and CRE, increasing trends have been shown in human setting (Swedres-Svarm, 2020). In companion animals and livestock, except for broilers and laying hens, prevalence of ESBL and pAmpC-producing *E. coli* has remained low, and no carbapenemase-producing Enterobacteriaceae (CPE) has been detected (Swedres-Svarm, 2020). Today, the occurrence of ESBL/pAmpC-producing *E. coli* in broilers is also low but there was a high occurrence in 2010 to 2017 linked to introduction by and transmission from contaminated imported breeding stock (Nilsson et al., 2020). Occurrence of *mcr*-genes in Sweden appears uncommon with only a handful human cases and no cases in animals, except for *mcr*-9 which has been identified in clinical colistin susceptible ESBL-producing Enterobacteriaceae isolates from horses (Börjesson et al., 2020; Swedres-Svarm, 2020).

A recent EFSA report (EFSA, 2021) highlighted that feed can be contaminated by a range of resistant bacteria and is therefore a potential route for introduction of antibiotic resistant bacteria to the livestock population. Furthermore, as demonstrated by Crump et al. (2002), colonization and infections of Enterobacteriaceae in food-producing animals and outbreaks of *Salmonella* in humans can also be traced back to contaminated feed. Particularly imported soy products have been pointed out as high-risk (Wierup, 2017). In Sweden feed materials with high protein content, such as soya, are mainly imported from European countries, e.g., Italy or third countries like Brazil, India and China (Statistics Sweden, 2022). As ESBL-, pAmpC-producing Enterobacteriaceae, CRE and Enterobacteriaceae with *mcr*-genes are commonly detected in different compartments in these geographical areas, import of feed products from there might represent a potential introduction route for these bacteria to the Swedish livestock population (Sun et al., 2017; Li et al., 2019; García-Betancur et al., 2021; Lalhruaipuii et al., 2021). However, studies about the role of feed in spreading transferable resistance to extended spectrum cephalosporins, carbapenems or colistin in Enterobacteriaceae in the feed chain is lacking.

The aim of the present study was to investigate if *Escherichia* spp., *Enterobacter* spp., *Klebsiella* spp., or *Raoutella* spp. with transferable resistance to extended cephalosporins, carbapenems or colistin could be detected in the environment of feed material intake in Swedish feed mills. Any identified strains were characterized genotypically and phenotypically, and their relatedness to previously described isolates from animal and human sectors in Sweden was thereafter investigated.

## Materials and methods

### Feed mills and sampling

The present study included all Swedish feed mills (*n* = 25) producing compound livestock feed in 2019. According to the Swedish *Salmonella* control program, feed mills are obliged to submit weekly environmental samples collected from predetermined sample points (National Veterinary Institute (SVA), 2022). The study utilized environmental samples from the feed material intake, pit or bottom part of the elevator for feed materials at the feed mills. Dust was collected with a swab (dry or wet) or scraped off, the sampling method varied between feed mills. During the autumn of 2019, four or five environmental samples from the feed material intake were collected with about a month apart, from each of the feed mills, and included in the current study. In total 113 environmental samples were included, and all samples were anonymized for this study.

### Isolation and identification of carbapenem, third-generation cephalosporin and colistin resistant *Escherichia* spp., *Enterobacter* spp., *Klebsiella* spp., and *Raoutella* spp.

**Figure 1:**
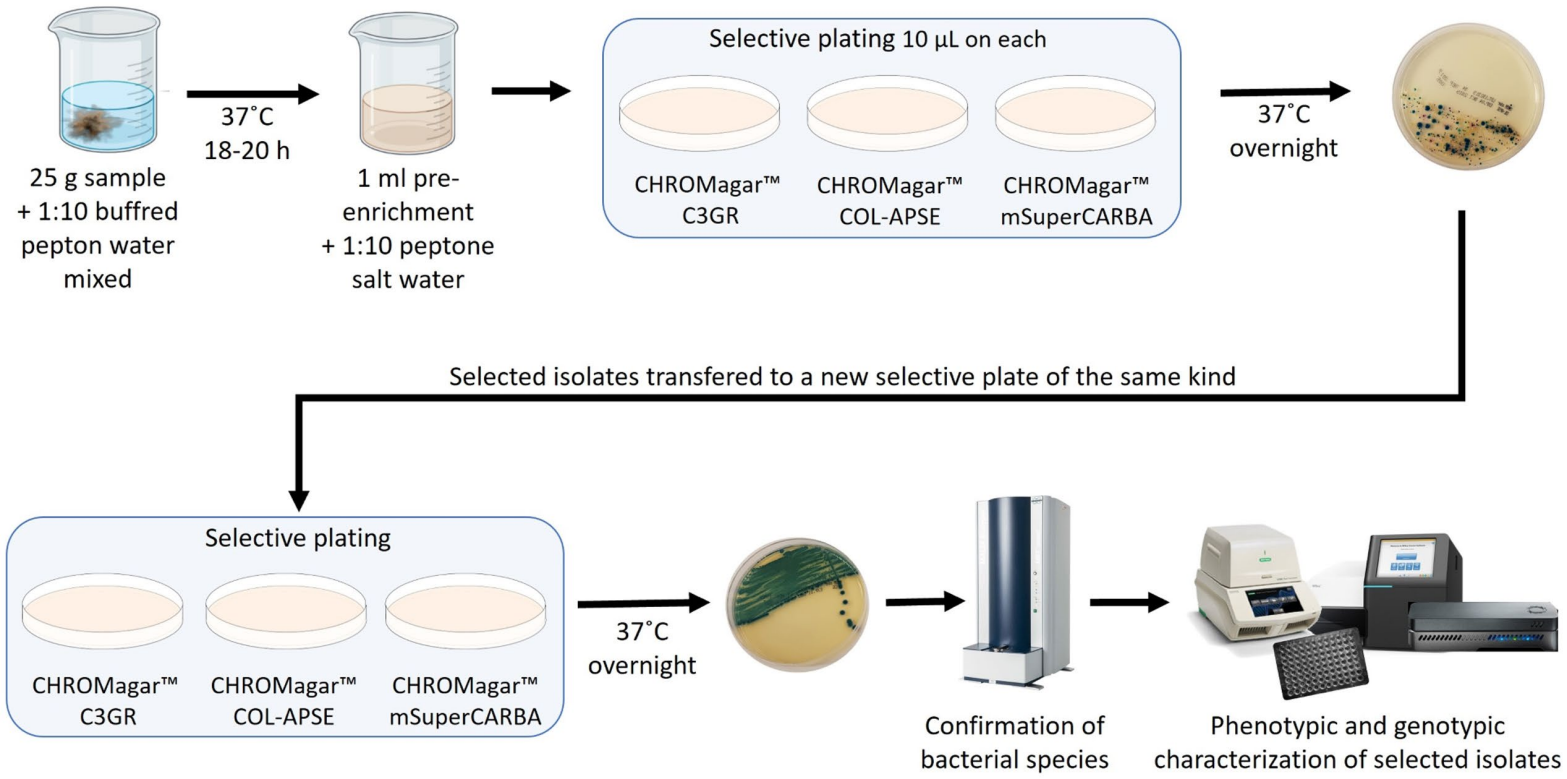
Schematic outline of the study.

From each environmental sample 25 g material was collected and then diluted 1:10 in Buffered Pepton Water (BPW). The 25 g material and BPW was mixed with a spoon and incubated at 37°C for 18–20 h as shown in Figure 1. One ml of the pre-enrichment was then diluted 1:10 with Peptone salt water, and from this dilution 10 μl was transferred to a CHROMagar^™^ C3GR, CHROMagar^™^ mSuperCARBA^™^ and CHROMagar^™^ COL-APSE plate, respectively, and the agar plates were incubated at 37°C overnight. Suspected colonies were identified based on the colony morphology as described by the manufacturer, and sub-streaked on the same selective agar again. Bacterial species identification was performed using a Bruker Biotyper MALDI-TOF MS.

### Phenotypic and genotypic characterization of isolated *Escherichia* spp., *Enterobacter* spp., *Klebsiella* spp., or *Raoutella* spp.

*Escherichia* spp. *Klebsiella* spp., or *Raoutella* spp. isolates growing on C3GR were subjected to multiplex-PCRs detecting the gene groups: pAmpC (Pérez-Pérez and Hanson, 2002), *bla*_CTX-M_ (Woodford et al., 2005), *bla*_SHV_, *bla*_TEM_ and *bla*_OXA-1_ (Fang et al., 2008), and *Enterobacter* spp. isolates were subjected to the same PCRs except for the pAmpC PCR. Isolates belonging to the same species growing on the COL-APSE were subjected to PCR detecting the genes *mcr*-1 to *mcr*-5 (Rebelo et al., 2018). Isolates from the mSuperCARBA were tested for carbapenemase-production using the RAPIDEC^®^ CARBA NP (bioMérieux SA).

If shown to carry transferable genes encoding ESBL, AmpC, colistin resistance or had carbapenemase activity these were checked for antibiotic susceptibility using Sensititre™ EUVSEC microdilution panels (Thermo Fischer Scientific), and any isolate with reduced susceptibility to colistin were also re-tested using MICRONAUT MIC-Strip Colistin (Merlin Diagnostika) according to the recommendations of the manufacturers. Isolates were determined as resistant based on EUCASTs Epidemiological cut-off value (ECOFF) (EUCAST, 2022).

A single isolate designated SB89A was positive for carbapenemase-production and resistant to carbapenems and colistin and was therefore subjected to short-read sequencing. The isolate was cultured on horse blood agar from which bacterial material was collected and used for DNA extraction with the EZ1 DNA Tissue Kit (QIAGEN, Germany) according to the manufacturer’s protocol. DNA concentrations were determined using Qubit^™^ HS DNA Kit (Life Technologies) and the DNA was used for library preparation with the Illumina Nextera XT Kit (Illumina Inc) and 250-bp paired-end sequencing was performed on an Illumina MiSeq sequencer (Illumina Inc.). These reads were assembled and checked for plasmid replicons and resistance genes as described in Appendix 1, using Trimmomatic, SPAdes, Pilon, ARIBA with species checked with Kraken and rMLST, and assigned a sequence type (ST) using multi-locus sequence typing (MLST).

### Plasmid characterization

The isolate SB89A which was shown to carry transferable genes encoding resistance to carbapenems was in addition to short-read sequencing also sequenced on an Oxford Nanopore MinIon, using the PCR barcoding kit SQK-LWB001 and a 9.4 MinION flowcell, producing reads with a median length of 2,686 bp. An initial hybrid assembly was performed with Unicycler (Wick et al., 2017) and annotated with Prokka (Seemann, 2014) which showed that the bla_IMI-2_ gene was located on a plasmid. However, this assembly did not produce a circularized sequence (details in Appendix 1). Plasmid DNA from SB89A isolate was therefore purified and transformed into a *E. coli* DH10B selecting for ertapenem resistance before the molecular size was determined using S1-restricted pulsed-field gel electrophoresis as previously described (Liakopoulos et al., 2016). Long-read sequencing on a MinION^™^ Flowcell FLO-MIN106D and using a Ligation sequencing kit SQK-LSK109 was performed for the wild-type isolate and the transformant *E. coli*, producing reads with a median length of 7,718 bp.

Long reads from the transformant and the previous short reads were used to create the second hybrid assembly with Flye (Kolmogorov et al., 2019) and Pilon (Walker et al., 2014). This assembly was typed with PlasmidFinder (Camacho et al., 2009; Carattoli et al., 2014) and annotated with Prokka (Seemann, 2014). Predicted protein sequences were also searched against the NCBI non-redundant protein (nr) database with blastp. The assembly was compared to previously characterized *bla*_IMI-2_ plasmids (Accession numbers KX868552, KY680213, and CP033468) and a *bla*_IMI-3_ carrying plasmid (KT780723) through sequence alignment and visualization with Mauve (Darling et al., 2004). Segments that had no similarity to these plasmids were searched against the NCBI nucleotide collection (nr/nt) database using blastn (Altschul et al., 1990). The plasmid was also analyzed with PHASTER (Arndt et al., 2016) to identify potential prophages. Analysis details including program versions and parameters are listed in Appendix 1.

### Comparison to isolates from other sources in Sweden

The Public health agency of Sweden (PHAS), previously the Swedish Institute for Communicable Disease Control, has since 2007 collected and verified all Enterobacteriaceae suspected of producing carbapenemases, and since 2016 all isolates have been genome sequenced using IonTorrent^™^ (Swedres-Svarm, 2020). As of 2010 the National Veterinary Institute (SVA) screens all samples collected from livestock for ESBL, pAmpc and carbapenemase producing *E. coli* within Svarm, and Swedish veterinary laboratories are encouraged to submit presumptive ESBL, pAmpC-producing and colistin-resistant Enterobacteriaceae and CRE for verification and characterisation at SVA. From these different collections three human clinical isolates were identified to be positive for the same gene encoding carbapenemase as the SB89A isolate. The identified isolates were reassembled using CLC Assembly cell, and the assemblies used for species identification with rMLST and MLST detection. Further, the assembled contigs were aligned towards the recovered plasmid from SB89A using minimap 2 with asm10 settings (Li, 2018). The alignment in SAM-format was then sorted, converted to bam-files, and matching contigs were extracted into fasta-format, using samtools, for downstream BRIG visualization (Li et al., 2009). The whole assembly was also analyzed using Platon and BAKTA to identify *bla*_IMI-2_ in each set of contig collections as well as confirm their classification as plasmids (Schwengers et al., 2020, 2021). These three isolates were then subjected long-read sequencing on a MinION^™^ R9.4.1 with libraries prepared using the SQK-RKB004 kit and Rapid Barcoding Kit SQK-RBK110.96, producing reads with a median length of 5,200 bp. Reads from Nanopore sequencing were assembled using a long-read only approach, using the pipeline trycycler as described in Appendix 1. Following this, the plasmid sequences were then extracted and typed, annotated and compared to pSB89A/IMI-2 as described in “Plasmid characterization.”

## Results

### Collected isolates and verification of phenotype and genotype

Out of the 113 collected samples growth was recorded on 117 C3G, 4 mSuperCARBA and 93 COL-APSE agar plates. In total, 466 isolates were collected from the agar-plates, 260 from C3GR, 6 from mSuperCARBA and 200 from COL-APSE. After species identification 235 isolates belonging to the investigated species were selected for further characterization: 143 from C3GR, 1 from mSuperCARBA and 91 from COL-APSE.

The most common identified genus was *Enterobacter* spp. (*n* = 201), followed by *Escherichia* spp. (*n* = 20) and *Klebsiella* spp. (*n* = 13) as shown in Table 1. The most common typed species from all the three different types of plates were the *E. cloacae* complex, 139 from C3GR, 1 from mSuperCARBA and 61 from COL-APSE (Table 1).

**Table 1:** Number of feed-mill samples with growth of investigated bacteria genera and identified bacterial species from the three agar plates.

	CHROMagar^™^ C3GR	CHROMagar^™^ mSuperCARBA	CHROMagar^™^ COL-APSE	Total
Number of samples with growth	85	1	61	
*Enterobacter cloacae* complex	139	1	61	201
*Escherichia coli*	0	0	4	4
*Escherichia hermannii*	0	0	16	16
*Klebsiella aerogenes*	4	0	0	4
*Klebsiella pneumoniae* complex	0	0	8	8
*Klebsiella oxytoca* ^*^	0	0	1	1
Total	143	1	90	234

* The Bruker Biotyper MALDI-TOF MS can have difficulties differentiating between *K. oxytoca* and *Raoultella ornithinolytica*, but in this study the isolate was reported as *K. oxytoca*.

All isolates from the C3GR and COL-APSE agar plates were negative for the investigated transferable resistance genes. The only isolate designated SB89A from the mSuperCARBA plate belonged to the *E. cloacae* complex and was shown to have a weak carbapenemase reaction on the CARBA-NP test. This isolate was resistant to cefepime, ertapenem, imipenem and meropenem, but was susceptible to ceftazidime and cefotaxime (Table 2). It was also resistant to colistin and was therefore re-tested using the MICRONAUT MIC-Strip Colistin and was determined to have a MIC of 64 μg/ml but skipped wells in the microdilution plate were also recorded and it was therefore retested three times, see Table 3.

**Table 2:** Antibiotic Minimum inhibitory concentrations (MICs), μg/ml, for the *E. cloacae* complex SB89A isolate using Sensititre^™^ EUVSEC and EUVSEC2 panels and *E. coli* transformant T9.2-pSB89a using EUVSEC3 and EUVSEC2 panels. Bold MIC indicates MIC above ECOFF for *E. cloace* or *E. coli*

Antibiotic	*E. cloacae* complex SB89A	*E. coli* transformant T9.2-pSB89a
Ampicillin^*^	> 64	**> 32**
Azithromycin^*^	8	4
Cefotaxime	0.5	≤ 0.25
Cefotaxime + Clavulanate acid	4	0.12
Ceftazidime	1	1
Ceftazidime + Clavulanate acid	2	0.5
Cefepime	**0.5**	0.25
Cefoxitin^*^	> 64	4
Chloramphenicol^*^	≤ 8	≤ 8
Ciprofloxacin	0.03	≤ 0.015
Colistin	**> 16**	≤ 1
Ertapenem	**> 2**	**> 2**
Gentamicin	≤ 0.5	≤ 0.5
Imipenem	**> 16**	**16**
Meropenem	**> 16**	**16**
Nalidixic acid^*^	≤ 4	≤ 4
Sulfamethoxazol^*^	≤ 8	≤ 8
Temocillin^*^	8	**32**
Tetracycline	≤ 2	≤ 2
Tigecycline	≤ 0.25	≤ 0.25
Trimethoprim	0.5	≤ 0.25

^*^ECOFF missing for *E. cloacae*.

**Table 3:** Colistin MICs (μg/ml) for the *E. cloacae* complex SB89A isolate using MICRONAUT MIC-strip Colistin and skipped wells.

Retest	1st	2nd	3rd	4th, from 3rd retest bacterial suspension^*^	
				4 μg/ml	16 μg/ml
MIC (μg/ml)	64	4	32	4	0.25
Concentration at skipped wells (μg/ml)	0.25 16	**–**	8	–	–

* Cultivated on blood agars plates and was confirmed as pure cultures and belonging to *E. cloacae* complex based on Maldi-TOF.

### Genotypic characterization of the SB89A isolate

The *E. cloacae* complex isolate SB89A was determined to be an *E. asburiae* carrying the *bla*_IMI-2_ gene, which encodes a carbapenemase, on a plasmid. The isolate was also positive for the intrinsic *bla*_ACT_ gene, the *fos*A gene conferring resistance to fosfomycin, and *oqx*B part of the OqxAB efflux pump which has been linked to reduced susceptibility to fluoroquinolones. In the SB89A the plasmid replicons IncFIB and IncFII were detected and based on the 7-MLST scheme for *E. cloacae* it belonged to ST657.

### Characterization of the plasmid carrying the *bla*_IMI-2_ gene

As initial hybrid assemblies of the Illumina and MinION data using different parameters resulted in potential plasmids of different sizes, PFGE of the wildtype SB89A isolate and an ertapenem-resistant *E. coli* transformant were therefore compared. While the SB89A isolate contained three independent plasmids, approximate 50, 150, and > 200 kb in sizes, the transformant only contained a single plasmid of approximately 200 kb in size, which validated the assembly of a 216,086 bp circular plasmid containing both IncFII(pECLA; 97.46% identity) and IncFIB (pENTAS01; 99.11% identity) replicons, here after referred to as pSB89A/IMI-2. In addition to the identified IncFII and IncFIB it contained a likely third replicon which showed 93.45% identity to IncFII(Yp). The phenotype of the *E. coli* transformant was confirmed using broth microdilution, showing that acquisition of the plasmid led to resistance against ertapenem, imipenem, meropenem, and ampicillin (Table 2).

**Figure 2:**
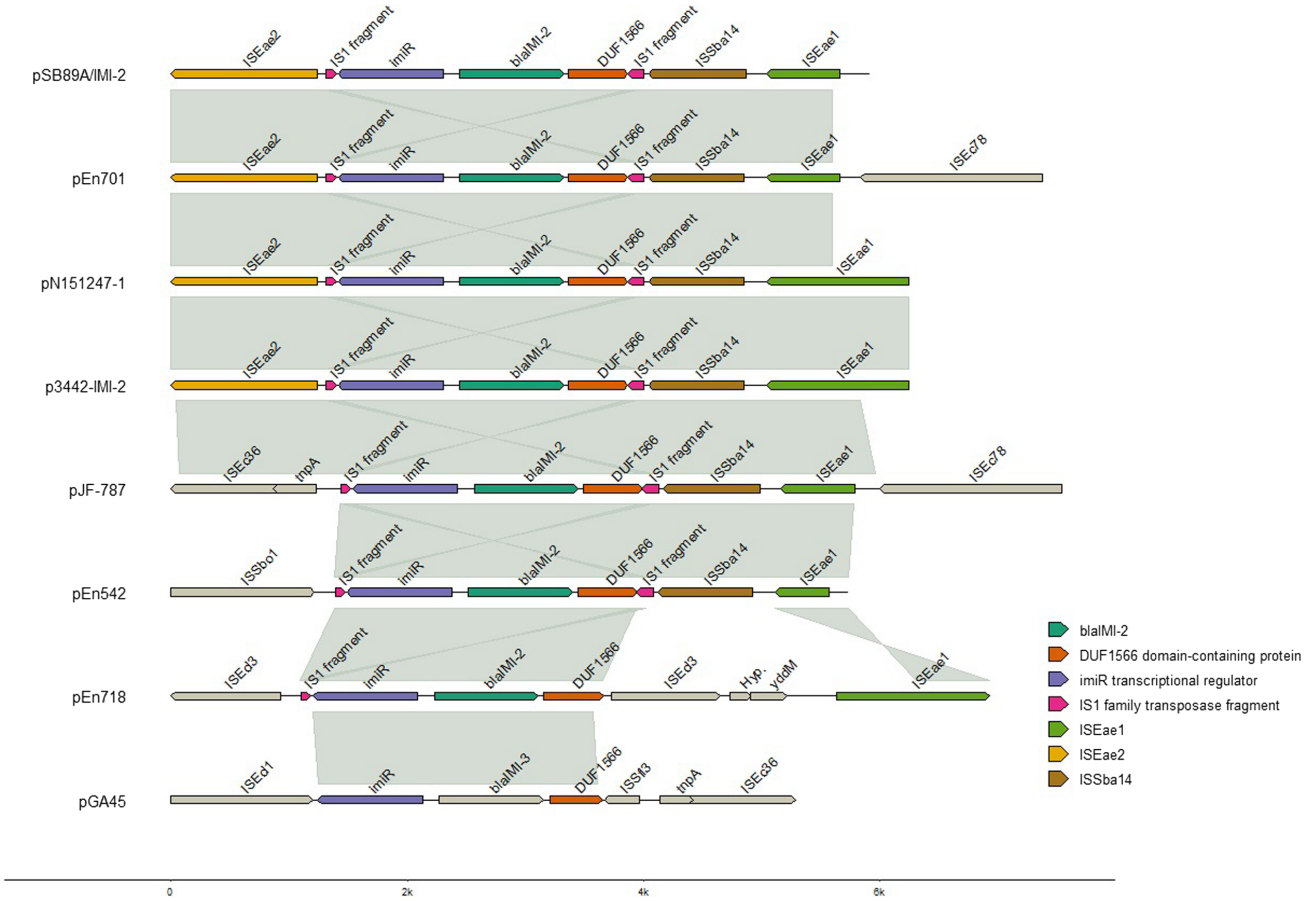
Alignment of *bla*_IMI-2_ genes and adjacent insertion elements. The aligned segments connected with gray lines shows 96.98–99.98% sequence identity, with the exception of p3442-IMI-2 and pJF-787 which showed 92.1% sequence identity. Figure created with genomes in R. For visualization purposes the sequences of pSB89A/IMI-2, pN151247-1 and p3442-IMI-2 have been inverted. The pGAA45 which carries a *bla*_IMI-3_ gene is also included for comparisons, as for the pSB89A/IMI-2, pN151247-1 and p3442-IMI-2 the sequence of pGAA45 have been inverted.

On pSB89A/IMI-2 the *bla*_IMI-2_ gene was associated with the *lys*R-like transcription regulator *imi*R and several complete and partial Insertion Sequence (IS) elements (Figure 2). The plasmid also contains genes for various functions such as plasmid segregation and stability (*par*A, *par*B*-like*, *par*M, *xer*C, *xer*D, *sop*A, *sop*B, *klc*A), DNA replication (*umu*C, *umu*D, *din*B, *rep*A, *rep*B, *rep*E-like), plasmid transfer (23 *tra* genes and three *trb* genes) and arsenical transport (*ars*A, *ars*B, *ars*C, *ars*D, and *ars*R2). The plasmid also contained the plasmid stability system *rel*BE/*stb*DE, but the *par*ED1 toxin/antitoxin system was also identified. Furthermore, the plasmid contained the *asn*O genes which encodes asparagine synthetase, *bio*F which encodes 8-amino-7-oxononanoate synthase, and the *tua*B encoding teichuronic acid synthesis a gram-positive cell wall component.

**Figure 3:**
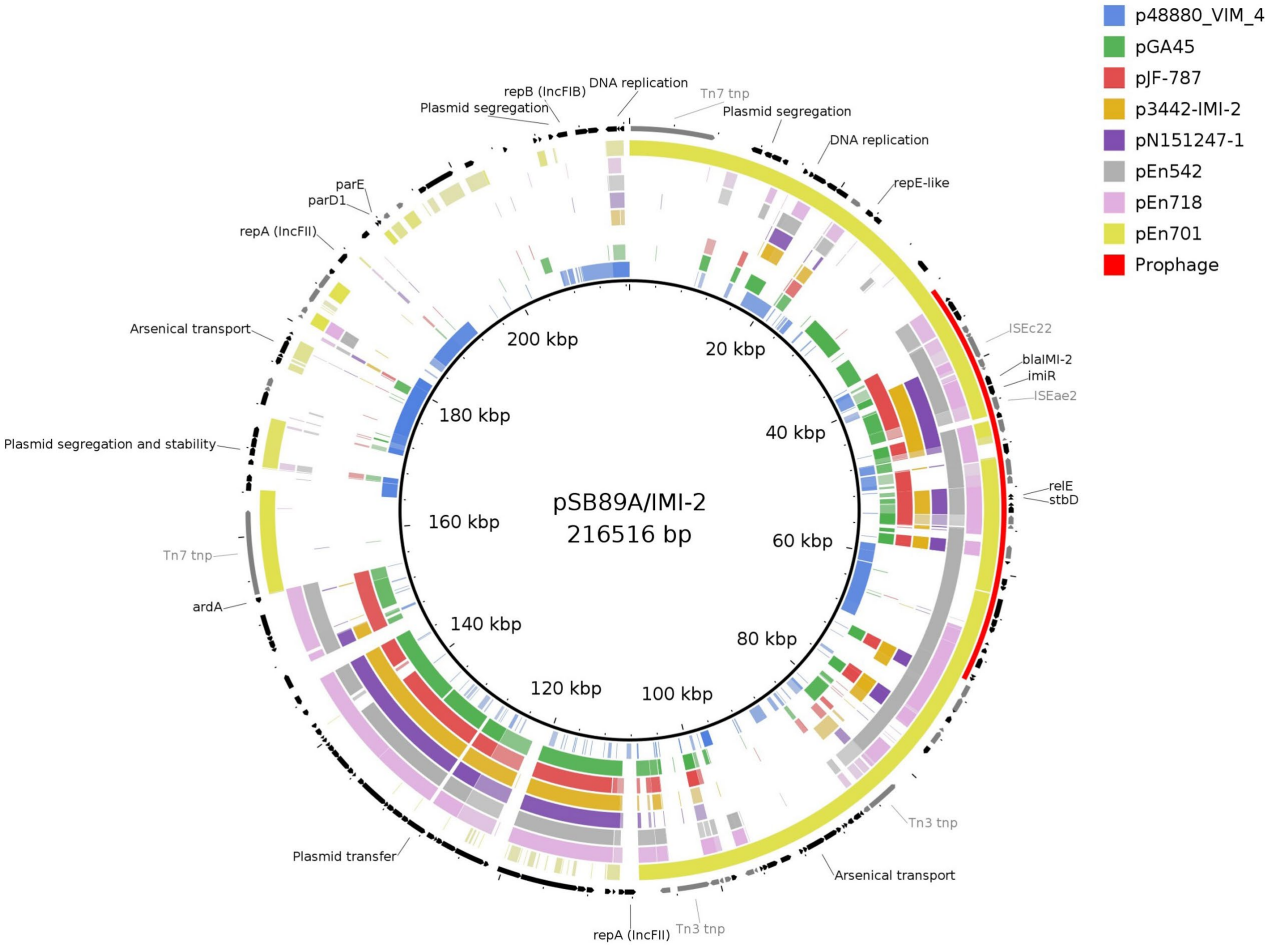
Comparison of pSBA89A/IMI-2 to three *bla*_IMI-2_ carrying plasmids from human clinical samples sequenced in this study, and to previously published plasmids of which three contains *bla*_IMI-2_ (KX868552, CP033468, and KY680213), one containing *bla*_IMI-3_ (KT780723), and one negative for *bla*_IMI_-genes (CP059418). Genes are shown in black and IS elements in gray. Figure created with BRIG.

PHASTER also identified a complete prophage (NC_049919) as part of the pSB89A/IMI-2 which spanned a region including the *bla*_IMI-2_ and the *teh*A gene which confers tellurite resistance (Figure 3). The pSB89A did not carry any additional genes encoding antibiotic resistance.

When comparing the pSB89A/IMI-2 with published *bla*_IMI-2_ plasmids it was shown that they shared the type IV secretion system for plasmid transfer as well as the composition of *imiR* and IS element genes surrounding *bla*_IMI-2_ (Table 4; Figures 2, 3).The IS*Eae*2 element downstream of *imiR* is an IS3-family insertion element similar to the IS*Ec*36 element described in other IMI-carrying plasmids (Dang et al., 2016). Upstream of the *imi*R and *bla*_IMI-2_ genes is a DUF1566 domain that codes for a protein of unknown function, and truncated sequences of transposases ISS*ba*14 and IS*Eae*1 which are also present in most of the compared plasmids (Figure 2). In two of the plasmids, pN151247-1 and p3442-IMI-2, the IS*Eae*1 sequence appears to be mostly complete (Figure 2). There are also small fragments of an IS1 family transposase present near *bla*_IMI-2_ in all compared plasmids. The pSB89A/IMI-2 plasmid also shared the DNA replication genes *umu*C and *umu*D with three of the compared plasmids. However, there are several sequence segments not shared among these plasmids including one containing an arsenical transport system, *rep*A, *rep*B plasmid segregation genes and IS elements flanked by *Tn7* transposons and one containing another arsenical transport system flanked by *Tn3* transposons. However, some of these sequence segments showed similarity to p48880_VIM_4 (CP059418), a *mcr*-9 and *bla*_VIM-4_ carrying IncHI2 plasmid (Bitar et al., 2020). When comparing the pSB89A/IMI-2 with a plasmid carrying the *bla*_IMI-3_ (pGA45) it was shown that it also had the DUF1566 domain-containing protein but shared none of the IS elements in the *imi*R – *bla*_IMI_ region with pSB89A/IMI-2 (Figure 2). It did however have an IS*Ec*36 IS-element present in the pJF-787 and highly similar in sequence to IS*Eae*2.

**Table 4:** Overview on plasmids sequenced in the current study and previously published plasmids containing *bla*_IMI-2_ (bold) and *bla*_IMI-3_ (underlined).

Plasmid	Replicon(s)	Size (kB)	Bacterial host	Source	Country	Year	References^*^
**pSB89A**	IncFII, IncFIB, IncFII(Yp)- group^**^	216	*E. asburiae*	Feed mill environment	Sweden	2019	This study
**pEn542**	IncFII(Yp)	181	*E. mori*	Human clinical	Sweden	2018	This study
**pEn701**	IncFII(Yp)	167	*E. ludwigii*	Human clinical	Sweden	2020	This study
**pEn718**	IncFII(Yp)	137	*E. mori*	Human clinical	Sweden	2020	This study
**pN151247-1**	IncFII(Yp)- group^**^	60	*K. aerogenes*	Shrimp imported from Bangladesh	Canada	Unknown	Unpublished NZ_KY680213
**p3442-IMI-2**	IncFII(Yp)	78	*E. cloacae complex*	White shrimp (*Litopenaeus vannamei*) imported from India	Netherlands	2017	Brouwer et al. (2019)
**pJF-787**	IncFII(Yp)	78	*K. variicola*	Human clinical	Wales, United Kingdom	2011	Hopkins et al. (2017)
pGA45	IncFII(Yp) group^**^	141	Unknown	Haihe River sediment	China	Unknown	Dang et al. (2016)

* If unpublished the accession number at the National Center for biotechnology information (NCBI) is given.

** Less than 95% similarity to reference.

### Genotypic comparison of SB89A and pSB89A/IMI-2 with human clinical isolates

Out of the 830 Enterobacteriaceae verified to carry a gene encoding carbapenemases at PHAS from 2015 to 2020 only seven isolates were identified to carry a gene belonging to the *bla*_IMI_ group, and out of these only three isolates were identified to carry the *bla*_IMI-2_ gene. The first human clinical isolate carrying a *bla*_IMI-2_ was identified in 2018 and was an *E. mori* (Isolate ID En542) and the remaining two isolates were identified in 2020 and being an *E. ludwigii* (En701) and an *E. mori* (En718). All three isolates from humans carried the *bla*_IMI-2_ gene on an IncFII(Yp) plasmid of sizes 181,051 bp, 167,344 bp and 137,148 bp, respectively (Table 4). These were compared to pSB89A/IMI-2 (Figures 2, 3). In addition to *bla*_IMI-2_, all plasmids shared the *imi*R gene except for the plasmid in En701 (pEn701) they were overall dissimilar. However, the plasmids identified in En542 (pEn542) and En718 (pEn718) did share the same transfer system as pSB89A/IMI-2, see Figure 3. The pEn701 was highly similar in gene content and it contained both the *rel*BE*/stb*DE and *par*ED1 toxins/antitoxins, but the content was arranged in a different order compared to the pSB89A/IMI-2. The IS elements surrounding *bla*_IMI-2_ in pEn701 were identical to pSB89A/IMI-2 with the exception that pEn701 also contains the IS*Ec*78 (Figure 2). The other two showed less similarity with the pEn542 in only sharing the upstream IS elements with the pEn718 only sharing the closest sequences and an inverted IS*Eae*1. The pEn701 did also belong to a different replicon type IncFII(Yp) and the plasmid transfer system was dissimilar. A putative prophage spanning the area including bla_IMI-2_ was identified on the pEn701 which shared the *bla*_IMI-2_ region and prophages specific genes with the prophage identified in the pSB89A/IMI-2 plasmid, but regions before and after the *bla*_IMI-2_ region were dissimilar (Figure 3).

## Discussion

The current study showed that Enterobacteriaceae with transferable resistance to extended cephalosporins, carbapenems and colistin are uncommon in the environment of Swedish feed mills, with only a single *E. asburiae* carrying the *bla*_IMI-2_ gene isolated. However, there was growth observed of the investigated genera on the plates selective for extended-spectrum cephalosporin resistance (C3GR) and for colistin resistance (COL-APSE). This was not unexpected on the C3GR agar as it is designed not to inhibit growth of AmpC beta-lactamase producing strains and that the investigated genera can all carry intrinsic genes encoding beta-lactamase activity which can facilitate growth. In fact, the most commonly detected genus among the false-negatives were *Enterobacter* spp. (Table 1) which all carry different AmpC intrinsically (Jacoby, 2009). Furthermore, it was not also unanticipated to identify false-negative growth on the COL-APSE either, as *Enterobacter* spp. also can show reduced susceptibility to colistin, which was also observed for the *E. asburiae* carrying the *bla*_IMI-2_ gene isolated in the current study (Landman et al., 2013; Band et al., 2016; Hong et al., 2018). Resistance to colistin and extended-spectrum cephalosporin among the investigated genera might also been influenced by chromosomal mutations which were not investigated. In addition, the false-negative growth might have been influenced by growth by bacteria (data not shown) commonly occurring in the environment, which are intrinsically resistant to the investigated antibiotics (Cantón, 2009; Cox and Weight, 2013; Gogry et al., 2021).

This is to our knowledge the first time a carbapenem resistant Enterobacteriaceae has been described in a feed mill and the first description of such a bacterium in an environment linked to animals in Sweden. When reviewing the available literature, Enterobacteriaceae with the *bla*_IMI_ gene group appear uncommon both among humans and domesticated animals globally, and the same is seen in Sweden where only seven human cases so far has been reported (Swedres-Svarm, 2020; Bonnin et al., 2021). Nevertheless, the *bla*_IMI-1_ was described already in 1996 in two *E. cloacae* from patients in US, with the oldest isolate being from 1984, and since then, 21 additional variants have been described (Rasmussen et al., 1996; Bonnin et al., 2021; National Library of Medicine (NIH), 2021). As in this study, most previous studies have detected the *bla*_IMI_ genes in isolates mainly belonging to the *E. cloacae* complex (Bonnin et al., 2021). In addition, most descriptions are related to the outer environment and not clinical isolates, and primarily from or connected to aqueous environments. In fact, the *bla*_IMI-2_ was also described in 1996 in an *E. asburiae* isolated from a US river and, as in this study, the gene was located on a transferable plasmid (Aubron et al., 2005). Three additional *bla*_IMI-2_ *E. asburiae* isolates from three distant rivers were identified in the study, and since then, the *bla*_IMI-2_ gene has been described in *E. cloacae* complex isolates from, Spanish river sediments and vannamei white shrimp (*Litopenaeus vannamei*) originating from India (Piedra-Carrasco et al., 2017; Brouwer et al., 2019). Additionally, human clinical cases with *E. cloaace* complex carrying the *bla*_IMI-2_ have been reported from Czech Republic (Rotova et al., 2017), China (Yun-Song et al., 2006), Austria (Hartl et al., 2019) and South Africa (Gqunta et al., 2015). In the case report from Austria it was also reported that the infection was developed after a thermal bath visit. In addition, a recent French study also described a case related to a near-drowning in a river (Laurens et al., 2018). That study was also able to isolate *E. asburiae* with a *bla*_IMI-2_ from the river water in which the near-drowning had occurred and showed that these isolates were closely related to the isolates from the patient. The *bla*_IMI-2_ in *E. cloacae* complex appears therefore mainly linked, directly or indirectly, to environmental samples and appears uncommon in clinical settings. Thus, it is likely that the environment is the natural reservoir for these bacteria and the mobile elements carrying the *bla*_IMI-2_. In our study the *bla*_IMI-2_ isolate from the feed mill sample was compared with two *E. mori* and one *E. ludwigii* carrying *bla*_IMI-2_ from human clinical cases, but there was no information available about any environmental connection for these patients. However, as *bla*_IMI-2_ is located on transferable plasmids there is still a risk that this gene could be transferred to other species, or strains of *E. cloacae*, more prone to cause infections and spread in clinical settings, although this appears to happen very infrequently and the reason for this remains to be further elucidated. Plasmids with *bla*_IMI-2_ have nonetheless been identified in clinical *E. coli* isolates in Spain and China, in *Klebsiella variicola* in the United Kingdom and in *Pseudomonas aeruginosa* in Spain (Rojo-Bezares et al., 2012; Hopkins et al., 2017; Zhang et al., 2017; Cabrera et al., 2022).

In the current study the *bla*_IMI-2_ was located on plasmids belonging to the IncFII-family in all four investigated isolates. This mirrors the results of earlier published studies where the *bla*_IMI-2_ appears exclusively linked to IncFII plasmids, primarily IncFII(Yp), including those where the *bla*_IMI-2_ has been identified in isolates belonging to the *E. cloacae* complex species and in other Enterobacteriaceae (Rojo-Bezares et al., 2012; Hopkins et al., 2017; Piedra-Carrasco et al., 2017; Zhang et al., 2017; Brouwer et al., 2019). We therefore compared the pSB89A/IMI-2 plasmid identified in this study to earlier published plasmids and although it was shown that they shared some similar traits, the IncFII-plasmids varied greatly in both gene content and size (Figures 2, 3; Table 4). The same was true when comparing the pSB89A/IMI-2 to the plasmids identified in the three Swedish human clinical isolates. In contrast to the other plasmids the pSB89A/IMI-2 also contained multiple IncF-replicons, was the largest in size and it also contained sequence segments which showed similarity to an IncHI2 plasmid p48880_VIM-4 carrying a *mcr*-9 and *bla*_VIM-4_ (Figure 3). However, the IS*Eae*1, or a truncated version of it, was identified on all investigated *bla*_IMI-2_ plasmids in the *bla*_IMI-2_*-imi*R region and all had the toxin/antitoxins *rel*BE/*stb*DE (Figures 2, 3). It was also shown that all four plasmids sequenced in this study had a variety of different genes which could possibly give the isolates fitness advantages in different environmental settings such as genes encoding amino acid productions, arsenic and metal resistance, but any function by these genes needs to be further investigated. The pSB89A/IMI-2 and the pEn701 showed the greatest similarity with most overlapping genes including that both carried the *par*ED1 toxin/antitoxin, in addition to the *rel*BE/*stb*DE (Figures 2, 3). These two plasmids also had an almost identical *bla*_IMI-2_*-imi*R region with an identical IS*Eae*2 identified, thus indicating a potential common origin (Figure 2). Of the previously published plasmids two also had highly similar *bla*_IMI-2_*-imi*R regions to pSB89A/IMI-2 and pEn701, with the difference being primarily in the IS*Eae*1 (Figure 2). Interestingly, both of these plasmids originated from South-Asia and were isolated from shrimps (Table 4). However, no information was available if the patient or the positive feed mill had any contact with South Asia. The *bla*_IMI-2_*-imi*R regions in the remaining plasmids showed higher dissimilarity and did not carry the IS*Eae*2 (Figure 2). The pGA45 and the *bla*_IMI-2_ plasmid pJF-787 did have an IS*Ec*36 which is related to the IS*Eae*2 present in most other *bla*_IMI-2_ plasmids, with the exception of the pEn542 and pEn718. So it is possible that the IS*Eae*2 family could play a role in the transposition of *bla*_IMI_ genes, and the study describing the pJF-787 also suggested that IS*Ec*36 could have an essential role in the dissemination of *bla*_IMI_ genes (Dang et al., 2016). IS*Eae*1was not present in pGA45 which is present in all *bla*_IMI-2_ plasmids included in the current study, so this insertion sequence likely plays an essential role in understanding the dissemination of the *bla*_IMI-2_. Unfortunately, not much appears known about this IS except its connection to the *E. cloace* complex and that it appears to be first detected in this complex.^1^ In Sweden, IS*Eae*1 has also been identified in connection with a pathogenicity island in a group B *Salmonella enterica* isolated from porpoise (*Phocoena phocoena*) (Sandholt et al., 2021).

The *bla*_IMI-2_*-imi*R region in pSB89A/IMI-2 and pEn701 was also located on a putative prophage. The same prophage was also identified in this study in the plasmid, p3442-IMI-2, isolated in Netherlands from an Indian vannamei white shrimp (Brouwer et al., 2019). Interestingly, similar prophages have previously been linked with the Shiga toxin Stx1 in *E. coli* (Ogura et al., 2015). In addition, the *teh*A gene which previously has been identified in the chromosome of *E. coli* was also found on the prophages in this study (Taylor et al., 1994; Turner et al., 1997). These results can indicate that prophages may play a role in the spread of *bla*_IMI_ genes in the natural environment. Although not extensively studied it has been shown that transduction can play a role in the dissemination of genes encoding resistance (Colavecchio et al., 2017; Kondo et al., 2021; Wendling et al., 2021). In the case of STEC/EHECs prophages play an important role in the transfer and expression of the *stx*-genes, but they also transfer other virulence factors, and have been described to increases fitness of the host (Bondy-Denomy and Davidson, 2014; Wendling et al., 2021). Despite the *bla*_IMI-2_ gene being both connected to prophages and transferable plasmids spread to other bacterial species than the *E. cloacae* complex isolates appears infrequent, as previously discussed. The exact reason for the limited transmission beyond environmental *E. cloacae* complex strains of the *bla*_IMI-2_ gene and its corresponding IncFII-plasmids needs to be elucidated. However, as most other narrow-hosts IncF-plasmids they are likely dependent on both host-encoded and self-encoded factors for replication and the toxin/antitoxin systems also likely influences the limited dissemination (Kopotsa et al., 2019; Fraikin et al., 2020).

In addition to being a CRE due to the *bla*_IMI-2_ the *E. asburiae* isolate from the feed mill also displayed a hetero-phenotype for colistin. The isolate was shown on several retests being sometimes resistant and sometimes susceptible to colistin, with a skip-well phenotype also being observed (Table 3). However, this sort of hetero-phenotype has previously been described in *E. cloacae* complex isolates and is likely related to a subpopulation with an increased expression of *arn*B and *ept*A (Landman et al., 2013; Band et al., 2016; Hong et al., 2018). These two genes are responsible for the modification of lipid A, the molecular target of polymyxins, and were also detected in the chromosome of the SB89A isolate. As both *arn*B and *ept*A are located on the chromosome and are not transferable, this phenotype was therefore not further investigated in the current study.

Even though uncommon, the present study shows that the CRE occurs in the environment of feed mills and therefore feed materials may be a potential source for introduction of CREs to livestock and humans. The impact of antibiotic resistant bacteria in feed materials is likely reduced by the heat treatment that most Swedish compound feed for livestock is subjected to. However, it has been described that heat treatment performed on feed does not eliminate all bacteria (Cox et al., 1983) and occurrence of antibiotic resistant Enterobacteriaceae has been described in processed feed (da Costa et al., 2007; Fraiture et al., 2021). Since feed cannot be excluded as a route of introduction it ought to be considered as a material to screen for antibiotic resistant bacteria.

## Conclusion

Screening of the environmental samples from the feed material intake at Swedish feed mills revealed that transferable resistance to extended spectrum cephalosporins, carbapenems or colistin has a very low prevalence. A single carbapenemase producing *E. asburiae* was isolated during the study and was shown to encode a *bla*_IMI-2_ carbapenemase gene on an IncFII plasmid that had similarities, but no direct molecular epidemiological links, to *bla*_IMI-2_ encoding plasmids from Swedish human clinical and global clinical and environmental isolates could be established. Thus, this and other studies indicate that the environment may be a reservoir for the *bla*_IMI-2_ gene and that this gene is present on a diverse set of IncF-plasmids.

## Data availability statement

The datasets presented in this study can be found in online repositories. The names of the repository/repositories and accession number(s) can be found at: https://www.ebi.ac.uk/ena, PRJEB53023.

## Author contributions

SB, JoE, and LE conceptualized the study with all authors contributing to the final design. SB, MB, JoE, and LE secured funding for the project. SB, MB, and JeE performed the laboratory work, with SB, MB, EÖ, and OK performing the bioinformatic analyses. SB, MB, EÖ, OK, and LE were responsible for the data curation. SB wrote the first draft of the manuscript after which all authors contributed text. SB and LE finalised the manuscript. All authors contributed to the article and approved the submitted version.

## Funding

This work was conducted in the framework of the Full Force project, supported by funding from the European Union’s Horizon 2020 Research and Innovation program under grant agreement no 773830: One Health European Joint Programme. In addition, internal funds were used at the National Veterinary Institute (SVA) in Sweden.

## Conflict of interest

The authors declare that the research was conducted in the absence of any commercial or financial relationships that could be construed as a potential conflict of interest.

## Publisher’s note

All claims expressed in this article are solely those of the authors and do not necessarily represent those of their affiliated organizations, or those of the publisher, the editors and the reviewers. Any product that may be evaluated in this article, or claim that may be made by its manufacturer, is not guaranteed or endorsed by the publisher.

## Footnotes

^1^ https://isfinder.biotoul.fr/
