# Supplementary material for: Detection of an IMI-2 carbapenemase-producing Enterobacter asburiae at a Swedish feed mill
Source: Front Microbiol. 2022 Oct 21;13:993454. doi: 10.3389/fmicb.2022.993454 (PMC9634252; doi:10.3389/fmicb.2022.993454)
Supplement: Supplementary file 1 [file Data_Sheet_1.docx]

**Appendix 1**

Short read analysis

To confirm bacterial species and check for potential contaminations, reads were checked using Kraken2 against the Standard Kraken 2 Database (Wood et al., 2019). Reads were trimmed with Trimmomatic 0.36 and genome assembly was performed using SPAdes v.3.11.1 with ‘--careful’ parameter (Bankevich et al., 2012) followed by Pilon v.1.22. To identify carriages of transferable genes encoding antibiotic resistance and plasmid replicon types, Antimicrobial Resistance Identification By Assembly (ARIBA) v.2.14.1 (Hunt et al., 2017) with downloaded databases of ResFinder and PlasmidFinder (27^th^ of January 2020) were used. Species identification was re-checked using Ribosomal Multilocus Sequence Typing (rMLST) and Seven-loci multi-locus sequence typing (MLST) was performed at PubMLST.org.

First long read sequencing and plasmid assembly

The long reads used in this hybrid assembly were sequenced on an Oxford Nanopore MinIon using the PCR barcoding kit SQK-LWB001 and a 9.4 MinION flowcell, and basecalled with guppy 3.2.8 fast basecalling implemented in MinKNOW. Short reads were trimmed with Trimmomatic v. 0.39 and long reads were trimmed with Filtlong v. 0.2.0. Several hybrid assemblies were created with this data, though the best result was achieved through mapping the short and long reads to the *Enterobacter asburiae* L1 chromosome (Accession number NZ_CP007546.1) using Bowtie2 v. 2.3.5 and assembling the unaligned reads with Unicycler v. 0.4.8. The assembly was annotated with Prokka v. 1.14.5 and visualized with Bandage v. 0.8.1 which showed that the *bla*_IMI-2_ gene was most likely located on a plasmid of approximately 200 kbp though the assembly graph was not fully resolved.

Plasmid assembly and analysis

Long reads were trimmed and down sampled with Filtlong v. 0.2.0 and assembled with Flye v. 2.8.1. The circular contig carrying the *bla*_IMI-2_ gene was identified with ResFinder v. 4.1 (Database accessed 2021-03-16). This contig was polished with Bowtie2 v. 2.3.5 and Pilon v. 1.23 in three rounds using the short reads from the original sample. The plasmid sequence was annotated with Prokka v. 1.14.5 and typed with PlasmidFinder v. 2.1 (Database accessed 2021-04-22). Predicted protein sequences were searched against the non-redundant protein sequences (nr) database using BLAST. The finished assembly sequence was aligned with Mauve Multiple Genome Aligner to several previously characterized *bla*_IMI-2_ and *bla*_IMI-3_ carrying plasmids (Accession nrs. KT780723, KX868552, KY680213, CP033468). Segments that had no similarity to these plasmids were searched against the NCBI nucleotide collection (nr/nt) database using blastn.

| **Program** | **Step** | **Version** | **Parameters** | **Citation** |
| --- | --- | --- | --- | --- |
| Trimmomatic | Trim short reads | 0.39 | ILLUMINACLIP:NexteraPE-PE.fa:2:30:10:2:true SLIDINGWINDOW:4:25 MINLEN:36 | (Bolger et al., 2014) |
| Filtlong | Trim long reads | 0.2.0 | -p 90 --target_bases 600000000 --min_length 1000 | (Wick, 2021) |
| Unicycler | Hybrid assembly | 0.4.8 | --mode normal | (Wick et al., 2017) |
| Flye | Long read assembly | 2.8.1 | --min-overlap 5000 | (Kolmogorov et al., 2019) |
| Bowtie2 | Mapping short reads to Flye assembly | 2.3.5 | -X 1000 | (Langmead and Salzberg, 2012) |
| Pilon | Correction of Flye assembly | 1.23 |  | (Walker et al., 2014) |
| Prokka | Assembly annotation | 1.14.5 |  | (Seemann, 2014) |
| PlasmidFinder | Plasmid typing | 2.1 | ID: 90 % Coverage: 60 % Database: “Enterobacterales” | (Camacho et al., 2009; Carattoli et al., 2014) |
| ResFinder | Resistance typing | 4.1 | ID: 80 % Min length: 60 % Database: “Other” | (Camacho et al., 2009; Zankari et al., 2017; Bortolaia et al., 2020) |
| Mauve | Alignment of plasmids | 2015-02-13 snapshot |  | (Darling et al., 2004) |
| BRIG | Plasmid figure | 0.95 |  | (Alikhan et al., 2011) |
| Bandage | Plasmid visualization | 0.8.1 |  | (Wick et al., 2015) |
| gggenomes | Gene alignment figure | 0.9.5.9000 |  | (Hackl and Ankenbrand, 2022) |
| Python |  | 3.6 |  | (Python Software Foundation) |
| R |  | 4.1.2 |  | (R Core Team, 2021) |

Long-read only assembly of human isolates

Raw rads from High Accuracy Basecalling using Guppy XXX were used as a starting point for a Trycycler assembly, in short; reads were filtred to have a minimum length of 1000bp and the read-set was filtered to only include the 95% best sequences (those with highest raw Q score) using filtlong (Wick, 2021). Using the subsample script of trycycler (Wick et al., 2021), the dataset was split into 12 datasets. Three assembly strategies were employed on these datasets, using Flye, Raven, and Miniasm+Minipolish. Following assembly, the assembled contigs from all 12 assemblies were clustered and visualised as a dendrogram. Assemblies were manually curated based, all included clusters and contigs were then reconciled, Multiple sequence alignment was performed and reads from the initial filtering were mapped towards the curated contigs. After this, consensus sequences were produced and polished using medaka. For full instructions see [Home · rrwick/Trycycler Wiki (github.com)](https://github.com/rrwick/Trycycler/wiki).

| **Program** | **Step** | **Version** | **Parameters** | **Citation** | **Comment** |
| --- | --- | --- | --- | --- | --- |
| Filtlong | Filtering | 0.2.1 | filtlong --min_length 1000 --keep_percent 95 input_reads.fastq.gz > reads.fastq | (Wick, 2021) |  |
| Trycycler | Subsampling | 0.5.3 | trycycler subsample --reads reads.fastq --out_dir read_subsets | (Wick et al., 2021) |  |
| Flye | Assembly 1 | 2.9 | flye --nano-raw read_subsets/sample_01.fastq --threads "$threads" --out-dir assembly_X && cp assembly_0X/assembly.fasta assemblies/assembly_0X.fasta && rm -r assembly_0X | (Kolmogorov et al., 2019) | Performed four times, for assembly 1, 4, 7 ,10. X represents assembly number in code. |
| Raven | Assembly 2 | 1.8.1 | raven --threads "$threads" read_subsets/sample_0X.fastq > assemblies/assembly_0X.fasta && rm raven.cereal | (Vaser and Šikić, 2021) | Performed four times, for assembly 2, 5, 8,11. X represents assembly number in code. |
| Miniasm+Minipolish | Assembly 3 | 0.1.3 | miniasm_and_minipolish.sh read_subsets/sample_0X.fastq "$threads" > assembly_0X.gfa && any2fasta assembly_0X.gfa > assemblies/assembly_0X.fasta && rm assembly_0X.gfa | (Wick and Holt, 2019) | Performed four times, for assembly 3, 6, 9 ,12. X represents assembly number in code. |
| Trycyler | Clustering | 0.5.3 |  | (Wick et al., 2021) |  |
| Trycyler | Reconcilidation | 0.5.3 |  | (Wick et al., 2021) |  |
| Trycyler | Multiple Sequence alignment | 0.5.3 |  | (Wick et al., 2021) |  |
| Trycycler | Partitioning of reads | 0.5.3 |  | (Wick et al., 2021) |  |
| Trycycler | Consensus | 0.5.3 |  | (Wick et al., 2021) |  |
| Medaka | Polishing | 1.6.0 |  | (Nanoporetech, 2022) |  |

Alikhan, N.-F., Petty, N.K., Ben Zakour, N.L., and Beatson, S.A. (2011). BLAST Ring Image Generator (BRIG): simple prokaryote genome comparisons. *BMC Genomics* 12(1)**,** 402. doi: 10.1186/1471-2164-12-402.

Bankevich, A., Nurk, S., Antipov, D., Gurevich, A.A., Dvorkin, M., Kulikov, A.S., et al. (2012). SPAdes: a new genome assembly algorithm and its applications to single-cell sequencing. *J Comput Biol* 19(5)**,** 455-477. doi: 10.1089/cmb.2012.0021.

Bolger, A.M., Lohse, M., and Usadel, B. (2014). Trimmomatic: a flexible trimmer for Illumina sequence data. *Bioinformatics* 30(15)**,** 2114-2120. doi: 10.1093/bioinformatics/btu170.

Bortolaia, V., Kaas, R.S., Ruppe, E., Roberts, M.C., Schwarz, S., Cattoir, V., et al. (2020). ResFinder 4.0 for predictions of phenotypes from genotypes. *Journal of Antimicrobial Chemotherapy* 75(12)**,** 3491-3500. doi: 10.1093/jac/dkaa345.

Camacho, C., Coulouris, G., Avagyan, V., Ma, N., Papadopoulos, J., Bealer, K., et al. (2009). BLAST+: architecture and applications. *BMC Bioinformatics* 10(1)**,** 421. doi: 10.1186/1471-2105-10-421.

Carattoli, A., Zankari, E., García-Fernández, A., Larsen, M.V., Lund, O., Villa, L., et al. (2014). In Silico Detection and Typing of Plasmids using PlasmidFinder and Plasmid Multilocus Sequence Typing. *Antimicrobial Agents and Chemotherapy* 58(7)**,** 3895-3903. doi: 10.1128/AAC.02412-14.

Darling, A.C.E., Mau, B., Blattner, F.R., and Perna, N.T. (2004). Mauve: Multiple Alignment of Conserved Genomic Sequence With Rearrangements. *Genome Research* July 2004(14)**,** 1394-1403. doi: 10.1101/gr.2289704.

Hackl, T., and Ankenbrand, M.J. (2022). *gggenomes: A Grammar of Graphics for Comparative Genomics* [Online]. Available: https://github.com/thackl/gggenomes [Accessed].

Hunt, M., Mather, A.E., Sánchez-Busó, L., Page, A.J., Parkhill, J., Keane, J.A., et al. (2017). ARIBA: rapid antimicrobial resistance genotyping directly from sequencing reads. *Microbial Genomics* 3(10). doi: https://doi.org/10.1099/mgen.0.000131.

Kolmogorov, M., Yuan, J., Lin, Y., and Pevzner, P.A. (2019). Assembly of long, error-prone reads using repeat graphs. *Nature Biotechnology* 37(5)**,** 540-546. doi: 10.1038/s41587-019-0072-8.

Langmead, B., and Salzberg, S.L. (2012). Fast gapped-read alignment with Bowtie 2. *Nature Methods* 9(4)**,** 357-359. doi: 10.1038/nmeth.1923.

Python Software Foundation *Python Language Reference, version 3.6* [Online]. https://docs.python.org/3.6/. [Accessed].

R Core Team (2021). *R: A language and environment for statistical computing* [Online]. R Foundation for Statistical Computing, Vienna, Austria. Available: https://www.R-project.org/. [Accessed].

Seemann, T. (2014). Prokka: rapid prokaryotic genome annotation. *Bioinformatics* 30(14)**,** 2068-2069. doi: 10.1093/bioinformatics/btu153.

Walker, B.J., Abeel, T., Shea, T., Priest, M., Abouelliel, A., Sakthikumar, S., et al. (2014). Pilon: An Integrated Tool for Comprehensive Microbial Variant Detection and Genome Assembly Improvement. *PLOS ONE* 9(11)**,** e112963. doi: 10.1371/journal.pone.0112963.

Wick, R.R. (2021). *Trycycler* [Online]. https://github.com/rrwick/Trycycler. [Accessed].

Wick, R.R., Judd, L.M., Gorrie, C.L., and Holt, K.E. (2017). Unicycler: Resolving bacterial genome assemblies from short and long sequencing reads. *PLOS Computational Biology* 13(6)**,** e1005595. doi: 10.1371/journal.pcbi.1005595.

Wick, R.R., and Menzel, P. (2021). *Filtlong* [Online]. https://github.com/rrwick/Filtlong. [Accessed].

Wick, R.R., Schultz, M.B., Zobel, J., and Holt, K.E. (2015). Bandage: interactive visualization of de novo genome assemblies. *Bioinformatics* 31(20)**,** 3350-3352. doi: 10.1093/bioinformatics/btv383.

Wood, D.E., Lu, J., and Langmead, B. (2019). Improved metagenomic analysis with Kraken 2. *Genome Biology* 20(1)**,** 257. doi: 10.1186/s13059-019-1891-0.

Zankari, E., Allesøe, R., Joensen, K.G., Cavaco, L.M., Lund, O., and Aarestrup, F.M. (2017). PointFinder: a novel web tool for WGS-based detection of antimicrobial resistance associated with chromosomal point mutations in bacterial pathogens. *Journal of Antimicrobial Chemotherapy* 72(10)**,** 2764-2768. doi: 10.1093/jac/dkx217.
